# Supplementary material for: An AI-driven fire risk forecasting framework for urban villages using IGWO-optimized LSTM with incremental learning
Source: PLoS One. 2026 Jun 2;21(6):e0350182. doi: 10.1371/journal.pone.0350182 (PMC13229321; doi:10.1371/journal.pone.0350182)
Supplement: S2 File — (DOCX) [file pone.0350182.s002.docx]

附录 城中村火灾指标风险调查问卷

尊敬的各位专家：

感谢您在百忙之中抽空完成本问卷。

本问卷意在对城中村各指标风险程度进行评估，请您根据相关专业知识及自身经验填写本问卷。本问卷结果仅代表您对于指标风险程度的评估意见，无对错之分，请放心如实填写。

基本信息

您的工作/研究领域：

您的工作/研究年限：

致灾指标风险评分表

请您根据案例城中村的实际情况，使用阿拉伯数字“0~100”对下列致灾指标进行评分，分值越大表示该指标的风险程度越高。

表 致灾指标风险评分表

| 指标 | 风险程度 |
| --- | --- |
| 电气线路故障 |  |
| 电器设备故障或使用不当 |  |
| 电动车故障或使用不当 |  |
| 燃油车故障或操作不当 |  |
| 生产操作不当 |  |
| 储物不慎 |  |
| 生产设备故障 |  |
| 余火复燃 |  |
| 照明、炉具故障及使用不慎 |  |
| 烧荒、野外生火不慎 |  |
| 人为纵火 |  |
| 自燃、雷击等 |  |

**Appendix: Urban Village Fire Risk Indicator Assessment Questionnaire**

**Appendix. Fire Risk Indicator Survey for Urban Villages**

Dear Expert,

Thank you very much for taking the time to complete this questionnaire.

The purpose of this survey is to evaluate the risk level of fire-related indicators in urban villages. Based on your professional knowledge and practical experience, please assess the risk level of each indicator listed below. The results of this questionnaire represent only your expert judgment on the relative risk level of each indicator. There are no right or wrong answers. Please complete the questionnaire honestly according to your professional assessment.

**Basic Information**

Field of Work / Research Area:

Years of Professional Experience:

**Fire Hazard Indicator Risk Scoring Table**

Please evaluate the risk level of the following fire hazard indicators based on the actual conditions of the urban village case. Use an Arabic numeral score between **0 and 100** to rate each indicator. A higher score indicates a higher level of fire risk associated with the indicator.

**Fire Hazard Risk Indicator Evaluation Table**

| **Fire Hazard Indicator** | **Risk Score (0–100)** |
| --- | --- |
| Electrical wiring faults |  |
| Electrical equipment malfunction or improper use |  |
| Electric vehicle malfunction or improper use |  |
| Fuel vehicle malfunction or improper operation |  |
| Improper production operations |  |
| Improper storage of materials |  |
| Production equipment malfunction |  |
| Rekindling of residual fire |  |
| Lighting or stove malfunction and improper use |  |
| Careless outdoor fire use or burning of vegetation |  |
| Arson |  |
| Spontaneous combustion, lightning, and other natural causes |  |

**Questionnaire Notes**

- The scoring range for each indicator is **0–100**.
- A higher score represents a **higher fire risk level** associated with the indicator.
- Please base your evaluation on **professional knowledge and practical experience** related to urban fire safety management.
