# Supplementary material for: An AI-driven fire risk forecasting framework for urban villages using IGWO-optimized LSTM with incremental learning
Source: PLoS One. 2026 Jun 2;21(6):e0350182. doi: 10.1371/journal.pone.0350182 (PMC13229321; doi:10.1371/journal.pone.0350182)
Supplement: Attachment — Submitted filename: Response to Reviewers.docx [file pone.0350182.s003.docx]

An AI-Driven fire risk forecasting framework for urban villages using IGWO-Optimized LSTM with incremental learning

Reviewer #1

1-The manuscript references (a) 101 recorded incidents across 55 villages in 2023, and also (b) a “targeted study over 30 days” selecting 100 villages, producing 100 samples with a 30-day time step . It’s unclear whether these are the same dataset, two datasets, or one derived from the other. Please provide a single end-to-end data flow diagram/table: data sources → feature construction → Bayesian output generation → train/test split (75/25), plus exact counts at each stage.

**Reply:** Response to Comment 1,

Thank you for this helpful comment. We addressed your comments in our paper.

In fact, two independent datasets are employed in the proposed framework for different purposes.

The first dataset consists of real fire incident records collected from 55 urban villages in Chaoyang District during 2023, including 101 fire incidents. These data are used to statistically analyze the distribution of fire causes and to construct the Bayesian network structure.

The second dataset is a questionnaire-based time-series dataset obtained from 100 representative urban villages over a 30-day observation period. Expert scoring is applied to evaluate the fire risk indicators, which are then used as inputs to the Bayesian network. Through Bayesian inference, the corresponding fire probability is generated and used as the target variable for training the IGWO-LSTM-IL prediction model.

To clarify the overall workflow and the relationship between the two datasets, a data processing flow diagram has been added to the revised manuscript (Fig.8). The corresponding explanation has also been included in **Section 3.3 “Risk Prediction and Analysis”.**

2-The authors describe online gradient descent updates (Eqs. 16–17) and include “validate model and monitor drift” in Algorithm 1, but key implementation choices are missing: learning-rate selection/schedule, update frequency (per sample? per batch?), drift detection method/threshold, and how you avoid catastrophic forgetting when the data distribution shifts. Even a concise “IL settings” table would materially improve reproducibility and credibility.

**Reply:** Response to Comment 2:

Thank you for this valuable comment. We agree that the original manuscript lacked sufficient implementation details regarding the incremental learning (IL) procedure.

In the revised manuscript, we have clarified the key design choices of the IL strategy. Specifically, we have added the following content to **Section Incremental Learning Strategy (line 268) as:**

“Specifically, in this study, the main settings are as Table 2 as:

(1) Learning rate: A small constant learning rate (1e-4) is used during incremental updates to ensure stable parameter adaptation.

(2) Update frequency: Model updates are performed in a mini-batch manner when new data become available.

(3) Drift detection: A performance-based drift detection mechanism is introduced. When the prediction error (RMSE) on recent data exceeds 110% of the historical average, the model update is triggered.

(4) Catastrophic forgetting mitigation: To alleviate forgetting, a small portion of historical samples is retained and jointly used with new data during fine-tuning.

Table 2: Configuration of the incremental learning framework

| Component | Setting / Description |
| --- | --- |
| Incremental learning strategy | Fine-tuning of pre-trained LSTM (no reinitialization) |
| Learning rate | 1e-4 (constant during incremental updates) |
| Optimizer | Adam |
| Update frequency | Mini-batch update when new data arrive |
| Batch size | 10 |
| Number of epochs | 5–10 per incremental update |
| Data used for update | New data + small portion of historical data (replay mechanism) |
| Replay ratio | ~20% of historical samples |
| Drift detection method | Rolling RMSE-based monitoring |
| Drift threshold | Update triggered when RMSE exceeds 110% of historical average |
| Validation strategy | Online evaluation on recent samples |
| Catastrophic forgetting | Mitigated via replay of historical samples during fine-tuning |
| Model initialization | Continue from previously trained model parameters |

”

3-The probability aggregation relies on incident-proportion-derived weights, while the parent-node states are expert-scored and mapped into a_{B_i}\in\left[0,1\right]. Please specify questionnaire design (items per indicator), scoring rubric, normalization/mapping into [0,1], inter-rater reliability (e.g., ICC), and how missing/uncertain assessments are handled. This is especially important because the Bayesian output becomes the target for the LSTM.

**Reply:** Response to Comment 3:

Thank you for this important suggestion. We agree that the original manuscript provided insufficient detail regarding the expert scoring process, which is critical for reproducibility as the Bayesian output serves as the target variable for LSTM training.

To address this, we have added a concise description of the fuzzy evaluation framework to **Section Case Study(line392)** in the revised manuscript:

" In this study, a fuzzy–Bayesian integrated framework was adopted to reduce subjectivity in expert judgment. Experts from relevant fields (fire rescue, emergency management, architecture, and safety science) evaluated each fire risk indicator using a 0–100 scale. Their assessments were mapped to triangular fuzzy numbers corresponding to five linguistic levels (Very Low to Very High). Expert weights (0.2 for fire rescue and emergency management experts, 0.1 for the architecture expert, and 0.3 for the senior safety science professor) were assigned according to domain relevance and experience. The weighted fuzzy assessments were aggregated using $\lambda$-cut sets ($\lambda$ = 0.1) and defuzzified using the integral value method to obtain crisp scores ($a_{B_i}$) within the interval [0,1]. These scores were then used as inputs to the Bayesian network. The detailed questionnaire design and scoring rubric are available in the public repository: https://github.com/handongli2019/Urban-Village-Fire-Risk-Prediction-Using-IGWO-Optimized-LSTM-with-Incremental-Learning

We believe this clarification sufficiently addresses the reviewer's concerns regarding questionnaire design, scoring rubric, normalization, and expert weighting, while maintaining conciseness in the main text. The detailed mathematical formulations are available in the Github for readers seeking further technical depth.

4-The authors report strong gains (e.g., RMSE/MAE/R² improvements vs baseline LSTM)
, but the “normalized R²” reporting is confusing and may be misinterpreted without a definition and formula. Consider adding (i) confidence intervals via repeated runs or bootstrapping, (ii) a clear description of how hyperparameter tuning is done for each model to ensure fairness, and (iii) additional baselines (e.g., GRU, ARIMA/Prophet, XGBoost on lag features). Also, the headline “92.57% reduction in mean squared error” should be backed by an explicit table showing MSE values (not only RMSE/MAE) and the exact evaluation split/seed.

**Reply:** Response to Comment 4:

Thank you for this valuable suggestion. We have revised the manuscript to improve clarity and methodological transparency.

In **Section Model Comparison (line 542)**, we have reviewed the manuscript and confirmed that all evaluation metrics (MSE, RMSE, MAE, and R²) are reported as standard definitions. Any ambiguous terminology has been clarified to ensure consistent interpretation.

In **Section Case Study (line 435)**, we added the following content:

“In the simulation, both the Grey Wolf Optimizer (GWO) and the Improved Grey Wolf Optimizer (IGWO) have a population size of 30, with a maximum iteration count of 10, and operate in a 3-dimensional search space. The key LSTM hyperparameters—number of hidden units, number of training epochs, and learning rate—are optimized using the GWO and IGWO algorithms. The batch size is set to 32 based on the dataset characteristics (number of features, time steps, and samples). To ensure robustness, each model was independently executed 30 times with different random initializations, and the average results are reported as the final prediction outcomes.

Regarding additional baselines such as GRU, ARIMA, or XGBoost, we acknowledge their value as forecasting benchmarks. However, the primary objective of this study is to evaluate the effectiveness of the proposed optimization framework (IGWO) and incremental learning strategy (IL) in enhancing LSTM performance. Therefore, our baseline comparisons are designed as controlled experiments (LSTM vs. GWO-LSTM vs. IGWO-LSTM vs. IGWO-LSTM-IL) to isolate and quantify the contribution of each proposed component. A comprehensive comparison with other forecasting models is an important direction for future research and has been noted in the Conclusion section”

In **Section Case Study (line 408),** we have added

“Samples 1–75 are used for training and samples 76–100. All reported results are based on strict out-of-sample testing (75/25 train/test split) to ensure the model’s predictive validity and generalization ability. ”

In **Section Model Comparison (line 542)**, we have added the following table:

**Table. Algorithms Comparison**

| Model | LSTM | GWO-LSTM | IGWO-LSTM | IGWO-LSTM-IL |
| --- | --- | --- | --- | --- |
| MSE | 0.0181 | 0.0086 | 0.0038 | 0.0017 |
| MAE | 0.1085 | 0.0654 | 0.0447 | 0.0336 |
| R^2^ | 0.0192 | 0.4633 | 0.7011 | 0.8665 |

And the explanation **(line 541)**:

“All reported metrics (MSE, RMSE, MAE, R²) are calculated exclusively on the out-of-sample test set as the following table.”

These revisions improve the rigor, reproducibility, and clarity of the experimental evaluation.

Reviewer #2

1-The paper’s title includes the word “forecasting”, but there is an absence of engagement with the forecasting research literature. Armstrong & Green’s 2018 “Forecasting methods and principles: evidence-based checklists” would be a good place to start.

**Reply:** Response to Comment 1:

Thank you for this constructive suggestion. We acknowledge that the manuscript’s title emphasizes “forecasting,” and we agree that greater engagement with the forecasting methodology literature would strengthen the theoretical positioning of the study. In the revised version, we have expanded the introduction and discussion sections to clarify the forecasting framework adopted in this study, and we have added relevant references, including Armstrong & Green (2018), to better align the manuscript with established evidence-based forecasting principles.

So the changes are added accordingly in the **introduction part (line 53)**

“Recent studies have also emphasized the importance of evidence-based forecasting principles in predictive modeling. Armstrong and Green [20] proposed forecasting method checklists to improve the transparency and reliability of forecasting practices. In addition, large-scale forecasting evaluations such as the M4 competition [21] highlight the importance of rigorous model comparison in time-series forecasting research.

[20]. Armstrong, J. S., and Green, K. C. (2018). Forecasting methods and principles: Evidence-based checklists. Journal of Global Scholars of Marketing Science, 28(2), 103–159.

[21]. Makridakis, S., Spiliotis, E., and Assimakopoulos, V. (2020). The M4 competition: 100,000 time series and 61 forecasting methods. International Journal of Forecasting, 36(1), 54–74. “

2-From the above reference, an index model or knowledge model approach to the problems that is the subject of the paper would be a good contender for out-of-sample predictive validity.

**Reply:** Response to Comment 2:

We appreciate this insightful suggestion regarding index models and knowledge-based models. The primary objective of this study is to develop a data-driven predictive framework based on Bayesian risk estimation and optimized deep learning. While index or knowledge models represent valuable alternative approaches for out-of-sample prediction, they fall outside the methodological scope of the present work.

In the revised manuscript, we have clarified the research positioning and emphasized that the proposed model is evaluated using strict out-of-sample testing to ensure predictive validity. We also acknowledge the value of index/knowledge-based approaches as a promising direction for future comparative research.

Specifically, **in the Conclusion section (line 579), we have added the following content:**

**“**A comprehensive comparison with other forecasting models is an important direction for future research.**”**

Additionally, we have clarified in **Section Case Study (line 408),** that “All reported results are based on strict out-of-sample testing (75/25 train/test split) to ensure the model’s predictive validity and generalization ability.”

3. The scientific method ([thescientificmethod.info](http://thescientificmethod.info/)) requires testing hypotheses against plausible alternatives, including naïve and sophisticatedly simple ones. This paper compares two related ML models. See above, and the following research note which shows that Google’s expensive and data hungry “Flu Trends” ML model was easily beaten by a one observation univariate model: [https://www.researchgate.net/publication/349518744_Forecasts_of_doctor_visits_for_flu_Simple_conservative_methods_beat_Google%27s_big_data_machine_learning_model_Previously_titled_Comparison_of_forecasts_of_weekly_weighted_average_US_percentage_of_docto](https://www.researchgate.net/publication/349518744_Forecasts_of_doctor_visits_for_flu_Simple_conservative_methods_beat_Google's_big_data_machine_learning_model_Previously_titled_Comparison_of_forecasts_of_weekly_weighted_average_US_percentage_of_docto)

**Reply:** Response to Comment 3:

Thank you for raising this important methodological question. The Google Flu Trends example is a powerful reminder that complex models do not always outperform simpler ones. We agree that comparing against the baselines is essential for validating the added value of any proposed model.

As noted in our response to Comment 2, the primary objective of this study is to evaluate the effectiveness of the proposed optimization framework (IGWO) and incremental learning strategy (IL) in enhancing LSTM performance. Therefore, our baseline comparisons were designed as controlled experiments (LSTM vs. GWO-LSTM vs. IGWO-LSTM vs. IGWO-LSTM-IL) to isolate and quantify the contribution of each proposed component.

We acknowledge that a comparison with simple forecasting methods (e.g., naïve forecast, historical average, or linear regression) would provide additional context. Accordingly, we have added the following statement to the **Conclusion section (line 579)**:

"A comprehensive comparison with other forecasting models, including simple baseline methods such as naïve forecast and historical average, is an important direction for future research."

4. Squared error measures and in-sample fit are not useful for assessing relative out-of-sample forecast accuracy (predictive validity).

**Reply:** Response to Comment 4:

Thank you for this important comment. We fully agree that out-of-sample validation is essential for assessing predictive validity, and that squared error measures alone are insufficient.

To address this, we have made the following revisions in the manuscript:

In **Section Case Study (line 408)**, we have clarified that all models are evaluated using a strictly separated train-test split (75%/25%), and the test set is not involved in any stage of model training or parameter tuning. The random seed is set to 42, and the exact data split (samples 1-75 for training, 76-100 for testing) is now specified to ensure reproducibility.

In **Section Model Comparison (line 541),** we have stated that “all reported metrics (MSE, RMSE, MAE, R²) are calculated exclusively on the out-of-sample test set.”

In **Section Model Comparison (line 542)**, we now explicitly report MSE values alongside RMSE and MAE in a table, directly supporting the claimed 92.57% MSE reduction.

**Table 5. Comparison of prediction performance for different models.**

| Model | LSTM | GWO-LSTM | IGWO-LSTM | IGWO-LSTM-IL |
| --- | --- | --- | --- | --- |
| MSE | 0.0181 | 0.0086 | 0.0038 | 0.0017 |
| MAE | 0.1085 | 0.0654 | 0.0447 | 0.0336 |
| R^2^ | 0.0192 | 0.4633 | 0.7011 | 0.8665 |

These revisions ensure that all performance comparisons are based on rigorous out-of-sample evaluation, addressing the reviewer's concern regarding predictive validity.

5-Table 2 purports to list fire risk factors. A little consideration suggests others that are likely to be relevant; e.g., the prevalence and density of combustible: building materials, building contents, vegetation; local climate factors and weather; prevalence and use of spark or heat producing tools and machinery.

**Reply:** Response to Comment 5:

We thank the reviewer for this valuable suggestion regarding additional potential risk factors. In this study, the fire risk indicators were constructed to focus on ignition-related (hazard-triggering) factors within urban village environments, which represent the primary objective of the proposed risk prediction framework. Therefore, the indicator system emphasizes core causative variables aligned with the study scope.

We acknowledge that other factors, such as building material density, vegetation distribution, climate conditions, and equipment-related ignition sources, may also influence fire risk. These variables are highly relevant in broader risk assessment contexts. However, incorporating all possible determinants was beyond the scope of the present study, which aims to establish and validate a predictive modeling framework rather than to construct a comprehensive national-level risk index.

To address this, we have made the following revisions in the manuscript:

**In Section Determination of fire risk factor indicators (line 347)**, we have clarified that “The fire risk indicators in this study were constructed to focus on ignition-related (hazard-triggering) factors within urban village environments, based on statistical analysis of 101 real fire incidents in Beijing.” This empirically grounded approach ensures that the indicator system emphasizes the core causative variables most aligned with the study scope.

**In the Conclusion section (line 576)**, we have added the following statement:

"Future research could expand the indicator system to include additional factors such as building material density, vegetation distribution, and climate conditions, and validate the model's generalizability to other cities with different regional characteristics."

Reviewer #3

1-Dataset Size and Selection Bias

The study uses data from 100 selected urban villages, reportedly divided into training and
testing sets (e.g., 75/25), with a 30-day time-step structure.
Please address the following concerns:
• Why is this sample size sufficient for training an LSTM-based time-series model,
which is known to be highly data-hungry and prone to overfitting?

**Reply:** Response to Comment 1:

We appreciate the reviewer’s concern regarding dataset size and potential overfitting. In this study, the dataset consists of 100 urban villages, each represented as a multivariate time-series sample with a 30-day temporal window and 12 features. Therefore, the dataset provides 3,000 temporal observations and the effective training information is derived from structured temporal sequences rather than independent static samples.

As in the revised manuscript in **line 358** “Therefore, the dataset provides 3,000 temporal observations”

The LSTM architecture used in this work is deliberately designed with controlled model complexity. Moreover, the model parameters are optimized using the proposed IGWO framework, and performance is evaluated on an independent test set (75/25 split). The consistent improvements across multiple evaluation metrics (MSE, RMSE, MAE, and R²), compared with all baseline models, demonstrate that the model does not exhibit observable overfitting behavior.

Additionally, the incremental learning mechanism further enhances model adaptability and reduces the risk of overfitting by continuously updating the model with new data.

2-What statistical or methodological justification supports the adequacy of this dataset size?

The adequacy of the dataset is supported by the following methodological considerations:

**Reply:** Response to Comment 2:

Thank you for this question. The adequacy of the 100-village dataset is supported by the following methodological considerations:

- The study adopts a supervised time-series forecasting framework, where temporal dependency is the primary learning objective. Each village provides a 30-day time series with 12 features, resulting in 100 × 30 = 3000 time steps of sequential information for the LSTM to learn from.
- The dataset includes multivariate features across time, increasing effective information density beyond the number of independent samples.
- Independent training and testing sets (75/25 split) are used to evaluate generalization performance, with results consistently demonstrating strong predictive accuracy on unseen data.
- Multiple repeated experiments (30 runs with different initializations) were conducted to ensure stability and robustness of the reported results.

These design choices collectively support the methodological suitability of the dataset for the proposed model.

3-How were the 100 villages selected, and what steps were taken to avoid selection bias?

**Reply:** Response to Comment 3:

Thank you for this important question regarding village selection and potential bias.

The 100 urban villages were selected based on available official records and data completeness criteria. The selection process followed objective inclusion principles rather than outcome-based sampling. Specifically:

- All villages with complete 30-day time-series data for all 12 fire risk indicators were included in the dataset.
- The sample covers villages across different geographical areas within Chaoyang District, including both high-density and relatively lower-density zones.
- No villages were excluded based on their historical fire incident rates or fire occurrence levels, ensuring that the dataset is not biased toward high-risk or low-risk areas.

Therefore, no post-hoc filtering was applied, and the dataset represents the available and qualified samples within the study scope. The potential limitation regarding geographical generalization beyond Beijing has been acknowledged in the revised manuscript i**n Section Conclusion (line 576)**:

"Future research could expand the indicator system to include additional factors such as building material density, vegetation distribution, and climate conditions, and validate the model's generalizability to other cities with different regional characteristics."

4-This issue directly impacts model validity and generalizability.

IGWO Algorithm Novelty and Parameter Specification

The proposed Improved Grey Wolf Optimizer (IGWO) introduces nonlinear convergence

factors and Gaussian mutation mechanisms.

Please provide:
• Explicit mathematical formulations for:
o Maximum convergence factor (α_max)
o Mutation probability (p_m)
o Mutation factor (f_m)
o Dynamic weighting scheme
• Justification for chosen parameter values
Hyperparameter Optimization Strategy

**Reply:** Response to Comment 4:

We thank the reviewer for the valuable comments regarding the IGWO formulation and parameter specification. In the revised manuscript, the complete mathematical expressions of the improved mechanisms have been clearly provided to ensure reproducibility.

We added the “The maximum convergence factor is set to the standard value α_max=2” in the **Improved Grey Wolf Optimizer (IGWO) section (line 174)**, consistent with the original Grey Wolf Optimizer framework. The convergence factor decreases adaptively with iterations to achieve a proper balance between exploration and exploitation.

Also in Section **Improved Grey Wolf Optimizer (IGWO) (line 181), “**the mutation probability is set to p_m=0.1, which is a commonly used default value in evolutionary optimization algorithms to preserve population diversity while avoiding excessive randomness. The mutation factor f_m is set to 0.1, which provides a moderate perturbation strength to balance exploration and convergence.” This value is commonly adopted in evolutionary optimization algorithms and ensures stable performance.

No additional dynamic weighting factor is introduced, as the combination of nonlinear convergence control and Gaussian mutation was found to provide stable and improved performance in preliminary experiments.

For the simulation setup, In **Section Case Study (line 435)**, we added the following content:

“In the simulation, both the Grey Wolf Optimizer (GWO) and the Improved Grey Wolf Optimizer (IGWO) have a population size of 30, with a maximum iteration count of 10, and operate in a 3-dimensional search space. The key LSTM hyperparameters—number of hidden units, number of training epochs, and learning rate—are optimized using the GWO and IGWO algorithms. The batch size is set to 32 based on the dataset characteristics (number of features, time steps, and samples). To ensure robustness, each model was independently executed 30 times with different random initializations, and the average results are reported as the final prediction outcomes.

The search space of LSTM hyperparameters was defined as follows: the number of hidden units was set within [16, 128], the learning rate within [0.0001, 0.01], and the number of epochs within [50, 200]. These ranges were selected based on common practices in deep learning applications and prior related studies. The IGWO algorithm was then employed to search within this bounded space to identify the optimal configuration. All comparison models adopted the same search ranges to ensure fairness.”

These settings have been clearly stated in the revised manuscript to improve methodological transparency and reproducibility.

5-IGWO is used to optimize LSTM hyperparameters. Please clarify:
• The exact search space and bounds for each hyperparameter (e.g., hidden units,
learning rate, epochs, batch size)

• IGWO population size and number of iterations

• Computational cost compared to baseline optimizers

Additionally, justify the selection of IGWO over established optimization frameworks such as Bayesian optimization, Optuna, grid search, or random search.

**Reply:** Response to Comment 5:

We appreciate the reviewer’s comment. The search space of the LSTM hyperparameters, the IGWO population size, and the maximum number of iterations have already been clearly specified in the manuscript. Specifically, the hidden units, learning rate, and epochs were optimized within predefined bounded intervals, and the IGWO parameters (population size = 30, maximum iterations = 10, batch size=32) were reported in the **Section Case Study (line 434)**. All comparative models adopted the same search ranges to ensure fairness and reproducibility.

The computational cost of IGWO is comparable to that of other population-based optimizers, as they share the same population size and iteration settings. Since all methods were evaluated under identical conditions, the comparison remains fair.

Compared with grid search or random search, IGWO provides adaptive global search capability and avoids exhaustive enumeration of the parameter space. Unlike Bayesian optimization frameworks, IGWO does not rely on surrogate modeling assumptions and has lower implementation complexity, making it suitable for nonlinear and high-dimensional optimization problems. Therefore, IGWO was selected as a balance between search efficiency and algorithmic simplicity.

6-Please clarify:

• Why no statistical significance tests (e.g., paired t-test, Wilcoxon signed-rank test) were conducted to support comparative claims

**Reply:** Response to Comment 6:

Thank you for this important comment regarding statistical significance testing.

To address this, we have conducted Wilcoxon signed-rank tests to compare the predictions of the proposed IGWO-LSTM-IL model against each baseline model (LSTM, GWO-LSTM, and IGWO-LSTM). The Wilcoxon signed-rank test is a non-parametric alternative to the paired t-test and is appropriate here as it does not assume normality of the error distribution.

We added the following content to section **Section Model Comparison (line 555):**

“To assess the statistical significance of the performance improvements, Wilcoxon signed-rank tests were conducted. The results confirm that the proposed IGWO-LSTM-IL model significantly outperforms all baseline models (p < 0.01 for all comparisons).”

Table 6. Wilcoxon signed-rank test results comparing IGWO-LSTM-IL with baseline models.

| Comparison | p-value |
| --- | --- |
| IGWO-LSTM-IL vs. LSTM | < 0.001 |
| IGWO-LSTM-IL vs. GWO-LSTM | < 0.001 |
| IGWO-LSTM-IL vs. IGWO-LSTM | < 0.01 |

Reviewer #4

This study presents an approach where hyperparameters for LSTM are optimized using GWO and LSTM parameters are determined using a derivative-based algorithm. The article requires some revisions.
1- Statistical tests should be used in the comparison.

**Reply:** Response to Comment 1:

Thank you for this important comment regarding statistical significance testing.

To address this, we have conducted Wilcoxon signed-rank tests to compare the predictions of the proposed IGWO-LSTM-IL model against each baseline model (LSTM, GWO-LSTM, and IGWO-LSTM). The Wilcoxon signed-rank test is a non-parametric alternative to the paired t-test and is appropriate here as it does not assume normality of the error distribution.

We added the following content to section **Section Model Comparison (line 555):**

“To assess the statistical significance of the performance improvements, Wilcoxon signed-rank tests were conducted. The results confirm that the proposed IGWO-LSTM-IL model significantly outperforms all baseline models (p < 0.01 for all comparisons).”

Table 6. Wilcoxon signed-rank test results comparing IGWO-LSTM-IL with baseline models

| Comparison | p-value |
| --- | --- |
| IGWO-LSTM-IL vs. LSTM | < 0.001 |
| IGWO-LSTM-IL vs. GWO-LSTM | < 0.001 |
| IGWO-LSTM-IL vs. IGWO-LSTM | < 0.01 |

2- The cell architecture of the LSTM used is given, but the general architectural structure of the connections between LSTM cells is not provided. What is a time step? It is not specified and has not been included as a hyperparameter. What is the number of hidden layers in LSTM? The number of hidden layers is not the same as the number of hidden layer units. If the number of hidden layer units was considered as the time step, why was the number of hidden layers not considered as a hyperparameter?

**Reply:** Response to Comment 2:

First, in this study, a single-layer LSTM network was adopted. Therefore, the number of hidden layers was fixed to one and was not treated as a hyperparameter. The term “hidden layer” in deep learning typically refers to stacked network layers. Since the network depth was intentionally kept constant to ensure structural consistency and fair comparison, only the training-related hyperparameters were optimized.

**To address this, we have added the following clarification in Section Case Study (line437):** “A single-layer LSTM network is adopted in this study, with the number of hidden layers fixed to one.”

Second, the time step refers to the length of the input sequence used to construct the time-series samples. In this work, a 30-day sliding window was employed during data preprocessing to generate the input sequences. The time step represents the temporal dimension of the input data rather than a trainable model parameter. Therefore, it was kept constant across all experiments and was not included in the optimization process.

Finally, it is important to distinguish between the number of hidden layers and the number of hidden units. The number of hidden units defines the dimensionality of the hidden state within the LSTM layer, whereas the number of hidden layers determines the network depth. In this study, the network depth was fixed to one layer, and only the number of hidden units, learning rate, and training epochs were optimized using IGWO.

3-PSO has been used in the training of LSTM in the literature, why was GWO training, i.e., optimizing the weights of LSTM, not considered?

**Reply:** Response to Comment 3:

We thank the reviewer for this insightful question. Although metaheuristic algorithms such as PSO have been used in the literature to optimize LSTM weights, such an approach significantly increases the dimensionality of the search space and the computational burden. In contrast, this study focuses on hyperparameter optimization because LSTM performance is highly sensitive to hyperparameter settings (e.g., number of hidden units and learning rate). Proper hyperparameter selection has a substantial impact on convergence behavior, generalization ability, and overall prediction accuracy. Therefore, the proposed GWO/IGWO framework was designed to optimize key hyperparameters rather than network weights. The LSTM weights were trained using the standard gradient-based backpropagation algorithm to ensure computational efficiency and stable convergence.

To clarify this design choice, we have added the following statement to **Section Case Study (line438):** “It should be noted that LSTM performance is highly sensitive to hyperparameter settings. While metaheuristic algorithms such as PSO or GWO can theoretically be used to optimize LSTM weights, such an approach would significantly increase the computational burden due to the high dimensionality of the weight space. Therefore, in this study, the IGWO algorithm is employed only for hyperparameter optimization, while the LSTM weights are trained using standard gradient-based backpropagation to ensure computational efficiency and stable convergence.”

4-The literature review on the training of LSTM should be strengthened.

**Reply:** Response to Comment 4:

Thank you for this suggestion. To strengthen the literature review on LSTM model training, additional references related to LSTM-based time-series prediction have been added in the Introduction section, including studies by Kong et al. (2019) and Qin et al. (2017). These works further demonstrate the effectiveness of LSTM architectures in complex temporal prediction tasks.

We added this updates in the introduction part **(line 53)**

“Recent studies have further demonstrated the effectiveness of LSTM-based models in complex time-series prediction tasks. For example, Kong et al. [16] applied LSTM networks for short-term load forecasting, while Qin et al. [17] proposed a dual-stage attention-based recurrent neural network for time-series prediction. However, LSTM still suffers from issues such as long training time, sensitivity to hyperparameters, and potential overfitting.

[16]. Kong, W., Dong, Z., Jia, Y., Hill, D. J., Xu, Y., and Zhang, Y. (2019). Short-term residential load forecasting based on LSTM recurrent neural network. IEEE Transactions on Smart Grid, 10(1), 841–851.

[17]. Qin, Y., Song, D., Chen, H., Cheng, W., Jiang, G., and Cottrell, G. (2017). A dual-stage attention-based recurrent neural network for time series prediction. Proceedings of the 26th International Joint Conference on Artificial Intelligence

(IJCAI), 2627–2633.”
